# Correction of serum NSE reference intervals includes the unidentified hemolysis sample: 1‐year data analysis from healthcare individuals

**DOI:** 10.1002/jcla.22997

**Published:** 2019-08-11

**Authors:** Erfu Xie, Wei Zhang, Huaguo Xu, Yun Ling, Qiaodi Zhang, Shiyang Pan

**Affiliations:** ^1^ Department of Laboratory Medicine First Affiliated Hospital of Nanjing Medical University Nanjing China; ^2^ National Key Clinical Department of Laboratory Medicine Nanjing China

**Keywords:** free hemoglobin, hemolysis, neuron‐specific enolase, reference intervals

## Abstract

**Background:**

Reference intervals (RIs) are important for interpretation of laboratory results. Neuron‐specific enolase (NSE) can be utilized to aid the diagnosis of various tumors. However, while red blood cells contain NSE αγ‐isozymes, unrecognized slight hemolysis will result in increasing of NSE levels in serum. The aim of this study was to correct the NSE RIs from healthcare individuals results which may have unidentified microhemolysis.

**Methods:**

A total of 15 047 healthy individuals undergoing regular health care were recruited to redefine the NSE reference interval according to the CA28‐A3 document. Volunteers with NSE level between 16.3 ng/mL and the upper limit of new RIs were performed venipuncture for NSE retest. Simultaneously, serum free hemoglobin (fHb) was performed with o‐tolidine test.

**Results:**

Reestablishment of NSE RIs is 0‐18.9 ng/mL, which is wider than 0‐16.3 ng/mL provided by the manufacturer. Seventy‐four volunteers with the NSE level between 16.3 and 18.9 ng/mL were performed venipuncture for NSE retest. The ratio of NSE level drop to normal is 85.1% (63/74) in the subsequent results; there are significant differences between the median NSE of two groups (18.15 *vs* 14.15 ng/mL). Subsequently, the fHb concentration of 22 healthy individuals from 74 individuals was measured; there are significant differences between the median fHb of two groups (58 vs 30 mg/L).

**Conclusions:**

Some specimens with slightly elevated NSE may be attributed to the unrecognized slight hemolysis. The correction RIs may be expected to decrease the abnormal NSE results.

## 1 INTRODUCTION

Reference intervals (RIs) are important for interpretation of laboratory results. Some factors such as the inaccurate and inappropriate utility of RIs will result in improper clinical decisions. To make suitable clinical decisions, it is necessary to have appropriate RIs and proper application, especially tumor markers. As a tumor and neuron injury marker, serum neuron‐specific enolase (NSE) is widely used in the diagnosis of neuroblastoma, small cell lung cancer (SCLC), as well as neuron injury.^1^, ^2^ NSE is largely derived from secretion of neurons and neuroendocrine cells.^3^

However, NSE is comprised of two isoforms, including αγ and γγ, and αγ‐isozyme was also reported in erythrocyte and platelet. Some physical or chemical conditions, which could damage erythrocyte and platelet in blood samples, will increase αγ‐isozyme in serum and increase the concentration of NSE.^4^ Moreover, specimens of patients with slight hemolysis which are hardly observed with the naked eye could be neglected. The elevation of serum NSE caused by unidentified hemolysis will lead to improper clinical decisions in this condition. To eliminate the interference, Verfaillie CJ and Tolan NV.^5^, ^6^ established the correction equation for NSE measurement in hemolyzed serum samples which need to manually measure the free hemoglobin concentration of each serum sample. It seems inconvenient and cost‐effective.

Different from previous research, we try to solve the problem of the correction of NSE RIs. It is well known that the determination of the RIs is generally referenced to CLSI C28‐A3.^7^ Following the guidelines using a nonparametric method, RIs are typically derived from the population who may be different from the patient population undergoing medical evaluation. Alternatively, the RIs can be provided by the manufacturer and then transfer to the laboratory user by validation process.^8^ The NSE reference interval provided by manufacturer is 0‐16.3 ng/mL, and it is applicable in our laboratory by the transferring and validation process. The elevation of serum NSE caused by unidentified hemolysis will be considered as abnormal results, which may affect the clinical decision‐making. Inspiration from several studies that have attempted to derive reference values from patient samples,^9^, ^10^ the alternative way is to re‐establish the NSE reference interval including these invisible hemolysis specimens.

The aim of this study was to correct the NSE reference intervals from healthcare individuals even contain serum sample may have hemolysis that is invisible to the naked eye. Simultaneously, it is explored that the NSE value exceeds the original reference intervals whether caused by undefined hemolysis.

## 2 MATERIALS AND METHODS

### 2.1 Study population

This research was a retrospective analysis of NSE samples from the healthcare individuals recruited from medical examination center of First Affiliated Hospital of Nanjing Medical University from January 2017 to December 2017. Individuals with any one of the following conditions were excluded: (a) higher Cyfra21‐1 or CEA; (b) abnormal chest X‐ray; (c) malignancy; (d) chronic diseases (eg, hypertension, diabetes mellitus, hepatitis); (e) abnormal liver and kidney functions tests and abnormal hematology tests; and (f) naked hemolysis. Informed consent was achieved according to the guidelines on the fields of medical ethics and research ethics.

### 2.2 Quantitation of NSE in serum

Fasting blood samples were drawn from healthcare individuals in the morning and transported to our laboratory within 3 hours after phlebotomizing. After centrifuging at 1600 *g* for 5 minutes, serum was separated and analyzed automatically. NSE test was measured by Roche Cobas e 601 electrochemiluminescence analyzer system and corresponding reagents. The parameters of analyzer system were set as follows: linear range was 0.05 ~ 370 ng/mL, with the analytical sensitivity for <0.05 ng/mL.

### 2.3 Define of serum NSE reference interval

According to CLSI C28‐A3 on how to establish a reference interval by Clinical and Laboratory Standard Institute (CLSI),^11^ the reference interval should be built on samples from healthy population according to appropriate inclusive and exclusive criteria. Reference interval is an interval between the reference upper limit and lower limit. It is designated as the interval between a certain percentages (generally 95%) of values of the health population (usually a reference group) fall into. For most researches, the 2.5th percentile and 97.5th percentile are used as the lower limit and upper limit defining the reference interval. In many cases, only one side of the range has the clinical significance, which is usually the upper limit (the 97.5th percentile). As for the reference interval of NSE, with no clinical significance of the lower limit, the 97.5th percentile as the upper limit was used.

### 2.4 Quantitation of free hemoglobin in serum

Measurement of free hemoglobin (fHb) in serum was performed with o‐tolidine test. The mechanism is that the ferroheme in hemoglobin has similar activity with peroxidase, which can oxidize benzidine to present green color initially and then purple color. The concentration of fHb can be analyzed using UV spectrophotometer and a reference sample of already known fHb concentration.^12^

### 2.5 Statistical analyses

STATA software 12.0 was used for all statistical analysis. For prospective analyses (dependent samples), quantitative variables were compared using ANOVA. Quantitative variables were compared between two groups of independent samples using Mann‐Whitney test for variables with non‐normal distribution.

## 3 RESULTS

### 3.1 Study population summary

In this period, a total of 21 354 healthy individuals came to hospital physical examination center of First Affiliated Hospital of Nanjing Medical University for healthcare examination and required NSE test. According to the previous excluded criterion, individuals who do not meet the requirements will be excluded. Consequently, 15 047 healthy individuals were included, with 8374 male individuals (age range: 14‐75 years) and 6673 female individuals (age range: 15‐77 years).

### 3.2 Reference intervals

Previous studies show that there was no statistical difference in NSE reference interval between sex and age.^13^ Therefore, the NSE reference intervals are not stratified according to the sex and age in this study. Considering the lower limit of NSE reference interval has no clinical significance, the 97.5th percentile upper limit as the reference interval was used. The reference interval of NSE from this study is 0‐18.9 ng/mL. However, the reference interval of NSE is 0‐16.3 ng/mL which is provided by the manufacturer and validated by our laboratory. The range of reference interval, which was established according to the physical examination center is higher than our previous transferring and validation reference interval. The possible reason for the reference interval widening is the unrecognized microhemolysis in the serum of healthcare individuals.

### 3.3 Comparison between the NSE retests result and the initial result in healthcare individuals

While 0‐16.3 ng/mL as RIs of NSE is used, the results from 16.3 to 18.9 ng/mL will be considered slightly elevated, wrong clinical decisions may be implemented. In order to validate the possible false high level in this population with an NSE level between 16.3 and 18.9 ng/mL, 74 volunteers from this population were randomly chosen. Repeated blood sampling by highly skilled technicians to ensure successful sampling process was carried out. As showed in Table 1, an NSE retest level <16.3 ng/mL was seen in 63 patients. Only 11 patients had NSE levels higher than the reference interval, the ratio of higher NSE level drop to normal is 85.1% (63/74). As NSE distribution from 74 volunteers of initial and retest results are non‐normal distribution by ANOVA, Mann‐Whitney analysis is used to compare between the two groups. The retest results were significantly lower than the initial results (*P* = .00).

**Table 1:** Initial neuron‐specific enolase (NSE) results and retest results in 74 healthy individuals (n = 74)

Groups	Incidence of higher NSE	NSE (ng/mL)		*P*
		Level range	Median level	
Initial results	100% (74/74)	16.30 ~ 18.90	18.15	.00
Retest results	14.8% (11/74)	3.80 ~ 22.60	14.45	

### 3.4 Serum free hemoglobin measurement

The fHb levels in the initial serum and the retest serum of 22 healthy individuals randomly chosen from the 74 individuals were measured. As fHb distribution from these healthy individuals of initial and retest results are non‐normal distribution by ANOVA, Mann‐Whitney analysis is used to compare between the two groups. As shown in Table 2, the media of fHb concentration were 58 and 30 mg/L, respectively, marking a statistically significant higher fHb level in the initial serum than the retest serum (*P* = .00), which indicated slight hemolysis is a possible cause for the initially high NSE levels. As a preanalytic factor that may be neglected, microhemolysis may have affected the establishment of our reference interval.

**Table 2:** Initial fHb measurement and remeasurements in healthy individuals (n = 22)

Group	NSE level range (ng/mL)	fHB (mg/L)		*P*
		Level range	Median level	
Initial results	16.58‐18.70	27 ~ 110	58	.00
Retest results	3.80‐12.92	15 ~ 52	30	

Abbreviation: NSE, Neuron‐specific enolase.

## 4 DISCUSSION

Herein, we describe the correction of NSE reference intervals derived from healthcare individual samples. The NSE reference interval established in our study differs from the previous study.^14^ The main reason is that the reference interval of previous study is established according to the strict rules of C28‐A3, but in our research, the data derived from healthcare individuals may include unrecognized slight hemolysis in the serum.

It seems that the reference interval we defined was not accurate on account of not strictly referring to the exclude criterion of C28‐A3 guideline. However, because of some tests which could be interfered by conditions we cannot identify, the preanalytic factors may be taken into account in the process of defining reference intervals. Moreover, there are several studies that have attempted to derive reference values from patient samples.^10^, ^15^ Therefore, the reference interval established by our study did take into account the possibility of slight hemolysis in some specimens. The reference interval we obtain is wider than the manufacturer provided. This further confirms that there may be unrecognized hemolysis in the specimens that we choose.

As is well known, hemolysis blood sample may affect the measurement of many tests, such as serum potassium, NSE and lactate dehydrogenase (LDH), etc^16^ The hemolytic samples can be regarded as unqualified specimens and rejected. The visible hemolysis samples can be identified by machine or naked eyes easily, but if slight hemolysis could not be identified, erroneous results may lead to wrong clinical decisions.

In the previous studies, many laboratory scientists have been concerned about the impact of hemolysis on NSE results and try to correct it in different methods. Verfaillie CJ et al^5^ revealed a linear correlation between the concentration of NSE and the hemolytic index (H, 1H = 0.621 μmol/L Hb) and propose using a generalized correction factor according to the degree of hemolysis present in the sample. To adjust the NSE concentration, a term equal to (H × 0.30 μg/L) will be subtracted from the measured NSE concentration. Whereas, there is almost a 2‐fold difference in RBC NSE concentrations between individuals, indicating that this generalized correction will have difficulties providing accurate results. Subsequently, Nicole V. Tolan et.al^6^ established viable correction formula which took notice of individualized both on the patient level, to correct for inter‐individual variability in RBC NSE content, and on the assay level, accounting for Hb‐mediated signal quenching. Their studies correct the influence of hemolysis on NSE through the establishment of an equation. In practice, the amounts of free hemoglobin in serum or hemolytic index need to measure, simultaneously. Slight hemolysis, beyond our naked eye, attributed to NSE increasing may be ignored. Thus, it is necessary to correct the slight hemolysis which may affect our clinical decision.

In our study, the reference interval that we developed is 0‐18.9 ng/mL. The reference intervals of NSE are defined by the data derived from healthcare individuals may include unrecognized slight hemolysis in the serum. However, the reference interval we used in daily work is 0‐16.3 ng/mL which is provided by the manufacturer and previously validated by our laboratory. The range of reference interval established according to the physical examination center is wider than our previous transformation and validation result. The possible reason for this reference interval varying is the unrecognized slight hemolysis in the serum of healthcare individuals.

In order to validate our possible speculation, we chose 74 volunteers of this population with an NSE level between 16.3 and 18.9 ng/mL and draw blood samples by skilled technicians to avoid the hemolysis in drawing blood process. The second NSE results below 16.3 ng/mL were showed in 63 patients; this further confirmed that the first results, which will decrease in second results, were affected by hemolysis. Moreover, we measured the fHb levels in the initial serum and the second serum of 22 healthy individuals randomly chosen from the 74 individuals. It is showed that slight hemolysis as a preanalytic factor may be neglected, which may be taken into consideration on the progress in the NSE reference interval definition.

In summary, by correction of the NSE reference interval for the slight hemolysis in the serum of which may lead to clinical wrong decision, we redefine the NSE reference interval which the recruitment samples may involve the unrecognized slight hemolysis according to the C28‐A3 guideline. This correction reference interval may have some shortcomings such as that the reference interval becomes wider, which may cause some patients to be unable to diagnose. The other is that the reference interval is only from a single‐center study. The multi‐center study will be taken into consideration in our future research. However, its advantages are also obvious. The correction may be expected to decrease the abnormal NSE test which may minimize the next healthcare costs. The reference interval is derived from the healthy individuals of our hospital, ongoing working at our hospital, assessing the clinical utility of this redefined reference interval in the prediction of neurological diseases.

## CONFLICT OF INTEREST

Authors do not have any conflict of interest to disclose.
